# Managing a Complex Case of Bipolar Disorder in a Patient With Recurrent Hospitalizations

**DOI:** 10.7759/cureus.64271

**Published:** 2024-07-10

**Authors:** Chetanna Okpaleke Amazu, Kashish Nathwani, Mariana Berwerth Pereira, Dianella Rente Lavastida, Jonathan M Parker

**Affiliations:** 1 Psychiatry, St. George's University School of Medicine, True Blue, GRD; 2 Psychiatry, American University of the Caribbean, Miramar, USA; 3 Psychiatry, Jundiaí Medical School, Jundiaí, BRA; 4 Neurology, St. George's University School of Medicine, True Blue, GRD; 5 Psychiatry, Jackson Behavioral Health Hospital/University of Miami Health System, Miami, USA

**Keywords:** bpd-1, psychiatry, case study, recurrent hospitalizations, bipolar disorder

## Abstract

Bipolar disorder type 1 (BD-1) is a complex psychiatric disorder characterized by recurrent episodes of mania and depression. While manic episodes typically present with classic symptoms such as impulsivity, elevated mood, and increased energy, atypical presentations are not as common and when encountered may pose diagnostic challenges. In addition, multiple previous hospitalizations can prove for a more nuanced case with a potentially worse prognosis. This clinical case study explores the atypical clinical presentation of a 22-year-old Hispanic male with BD-1 and discusses the challenges associated with the correct diagnosis and recognition of this disorder.

Typical BD-1 symptoms consist of depressive and manic episodes. The mania can encompass elevated mood, increased energy, racing thoughts, decreased need for sleep, grandiosity, and impulsivity. The typical depressive episodes consist of fatigue, low mood, loss of motivation, changes in appetite or weight, and even suicidal thoughts. Atypical symptoms consist of a mixture of both mania and depression at once, psychosis, present with seasonal patterns, anxious distress, catatonia, and rapid cycling of mood.

The patient, with a medical history of BD-1, anxiety, polysubstance abuse, and multiple inpatient psychiatric hospitalizations presented to the emergency department via involuntary hold due to threats of suicidal behavior. Upon arrival, he presented with a myriad of typical and atypical acute manic symptoms including severe agitation, disorganization, anxiety, pressured speech, and rapid mood cycling. Throughout his admission he demonstrated extreme episodes of agitation, making threats of physical violence towards staff, attempting self-injury, behaving violently towards others, and displaying impulsivity as well as grandiosity despite receiving his long-acting injectable neuroleptic medication just three weeks prior to his hospitalization. Scheduled medication treatment during his inpatient hospitalization included a combination of risperidone, thorazine, divalproex sodium, mirtazapine, clonazepam, and temazepam. This clinical case underscores the importance of recognizing both typical and atypical presentations of manic episodes in BD-1 as well as the challenges involved in the treatment of a patient with severe and refractory symptoms requiring frequent hospitalizations.

## Introduction

Bipolar disorder type 1 (BD-1), formerly known as manic-depressive illness, is a chronic psychiatric disorder that significantly impacts an individual's mood, energy levels, and daily functioning. BD-1 is distinguished by the occurrence of at least one manic episode, often accompanied by periods of depression or hypomania. In contrast to manic episodes, hypomanic episodes are less severe, as they do not lead to significant impairment in functioning, and thus do not require hospitalization. In addition to presenting with severe symptoms during manic episodes, BD-1 patients are also more likely to be incapacitated by depressive episodes. The disorder is associated with substantial morbidity, impaired quality of life, and a heightened risk of suicide. While the exact etiology of BD-1 remains elusive, a combination of genetic, neurobiological, and environmental factors is believed to contribute to its onset and progression [1].

Manic episodes, the hallmark of BD-1, are characterized by an elevated mood, increased energy, impulsivity, and impaired judgment. Individuals may experience a decreased need for sleep, engage in risky behaviors, and exhibit grandiosity in thoughts and behavior. Depressive episodes, on the other hand, manifest as persistent feelings of sadness, hopelessness, and a loss of interest in previously enjoyed activities. The cyclical nature of BD-1 results in significant variability in mood, often leading to diagnostic challenges and delays in appropriate treatment initiation [2].

Emerging evidence suggests that gender plays a crucial role in the clinical presentation and course of BD-1. While the literature on BD-1 and gender disparities in hospitalizations is extensive, recent findings suggest that, on average, men tend to experience a lower number of hospitalizations due to manic episodes compared to women. Understanding these differences is crucial for tailoring treatment approaches and optimizing outcomes for individuals with BD-1 [3].

## Case presentation

A 22-year-old Hispanic male with a history of BD-1 presented to the inpatient psychiatric unit with symptoms of acute mania. His mania was noted to be both typical and atypical in symptomatology. The patient initially presented to the medical hospital under an involuntary placement order and was seen by a psychiatry consult service which recommended direct transfer to the behavioral health hospital. Upon arrival at the psychiatric unit, he demonstrated symptoms of acute mania including severe agitation, disorganization, pressured speech, and anxiety. He also demonstrated a high level of verbal and physical aggression toward hospital staff. His affect was notably expansive and labile. On initial interaction with the patient, he was seen pacing the unit, repeatedly interrupting rounds, and becoming increasingly frustrated when boundaries for appropriate behavior were set. The patient would then promptly begin laughing and joking with other patients and engaging with the game on television, evidence of a highly labile affect. Upon initial interview, the patient was very erratic, demonstrating high-energy behaviors, and expressed belligerently that he did not need to be hospitalized. He denied any thoughts of self-harm or suicidal ideation. The patient was also grandiose, endorsing the delusion that he was immortal. He indicated that this was his 18th psychiatric hospitalization within the period of the last three to four months. He adamantly refused medication treatment, so a healthcare proxy was sought.

The patient's family was contacted for consent to administer medications in an inpatient setting. They also provided a more detailed history, disclosing that the patient was initially referred to the clinic due to their concern regarding verbal expressions of suicidal ideation, and subsequently placed under an involuntary hold order. The patient’s mother indicated that the patient was abusing his psychiatric medications at home, reportedly “snorting” them. She additionally endorsed that the patient was using MDMA, cocaine, and cannabis. The family was very concerned with his multiple admissions, and their overall inability to manage him at home due to aggressive behavior. Prior to his hospitalization, his outpatient psychiatric medication prescriptions included Divalproex Sodium 250mg BID, Benztropine 0.5mg BID, Carbamazepine 200mg BID, Buspirone 10mg TID, Mirtazapine 15mg, Sertraline 50mg, Quetiapine 200mg, and long-acting Palperidone, at 156mg/mL and 234mg/1.5mL. He received the second dose of this long-acting injectable three weeks prior to his hospitalization. We were unable to confirm which of these prescription medications he was actually taking. 

Initial medication treatment started during hospitalization included scheduled Risperidone, Divalproex Sodium, and Clonazepam, along with as-needed medications for severe agitation including a combination of Thorazine, Olanzapine, and Diphenhydramine (Table 1). On day 2 of admission the patient presented less labile, though still very expansive, grandiose, and with continued agitation. On day 2, Mirtazapine 15mg was added for symptoms of insomnia, in addition to Buspirone 15mg TID for anxiety. An EKG was done on day 5 and presented unremarkable. Although the chemicals he allegedly used were not analyzed, a toxicological examination revealed ethanol levels less than 10.

**Table 1:** Chronologic administration of medications PO: per os (by mouth). This abbreviation indicates that the medication is taken orally. IM: intramuscular. This abbreviation indicates that the medication is administered via injection into a muscle. BID: two times a day. This abbreviation indicates that the medication is taken twice throughout the day. TID: three times a day. This abbreviation indicates that the medication is taken three times throughout the day. mg: milligrams. This unit of measurement is used to denote the dosage of medications. PRN: when necessary. This abbreviation indicates that the medication was given when required for extenuating circumstances such as excessive agitation.

Treatment Day	Day 1: 10/12/23	Day 2: 10/13/3	Day 3: 10/14/23	Day 4: 10/15/23	Day 5: 10/16/23	Day 6: 10/17/23	Day 7: 10/18/23	Day 8: 10/19/23	Day 9: 10/20/23	Day 10: 10/21/23	Day 11: 10/22/23
Risperidone	1mg PO BID	1mg PO BID	2mg PO BID	2mg PO BID	2mg PO BID	2mg PO BID	2mg PO BID	2mg PO BID	2mg PO BID	2mg PO BID	2mg PO BID
Divalproex Sodium	500mg PO TID	500mg PO TID	500mg PO TID	500mg PO TID	500mg PO TID	500mg PO TID	500mg PO TID	500mg PO TID	500mg PO TID	500mg PO TID	500 mg PO TID
Clonazepam	1mg PO TID	1mg PO TID	1mg PO TID	1mg PO TID	1mg PO TID	1mg PO TID	1mg PO TID	0.5mg PO TID	0.5mg PO BID	0.5mg PO BID	0.5mg PO BID
Mirtazapine		15mg PO hs	15mg PO hs	15mg PO hs	15mg PO hs	15mg PO hs	15mg PO hs	15mg PO hs	15mg PO hs		
Buspirone		15mg PO TID	15mg PO TID	15mg PO TID	15mg PO TID	15mg PO TID	15mg PO TID	15mg PO TID	15mg PO TID		
Lithium					450mg PO BID	450mg PO BID	450mg PO BID	450mg PO BID	450mg PO BID	450mg PO BID	450mg PO BID
Chlorpromazine						50mg PO TID	50mg PO TID	25mg PO TID	25mg PO TID		
Temazepam						15mg PO hs	15mg PO hs	15mg PO hs	15mg PO hs	15mg PO hs	15mg PO hs
PRN Medications Given for Agitation:											
Olanzapine	10 mg IM		10 mg IM								
Diphenhydramine	50mg IM		50mg IM 50mg IM		50mg IM 50mg IM	50mg IM 50mg IM					
Lorazepam					2mg IM						
Chlorpromazine	50mg PO				50mg IM						

Throughout his hospital course, the patient remained very discharge preoccupied. Over the next several days, he also continued to be labile, impulsive, heavily agitated, and expansive, with manifestations of elevated energy. During some episodes of agitation, he was witnessed banging his head against the nursing station’s plexiglass and verbally threatening staff. The patient also reached a level of impulsivity on day 5 that was a safety concern, as he was seen charging towards the security doors, and then attempting to physically break through them and enter a neighboring unit. The patient himself recognized that these behaviors were detrimental, but only after the fact. PRNs were given for the episodes of agitation, as outlined in Table 1.

Due to these severe episodes of agitation, Lithium 450 mg TID was added as a second mood stabilizer on day 5 of hospitalization. Over the initial seven days of his hospital stay, the patient became calmer and less agitated but was notably sedated from his medication regimen, causing some medication doses to tapered down. On day 8, both Chlorpromazine and Clonazepam dozes were subsequently lowered (from 50mg to 25mg TID and 1mg to 0.5mg TID, respectively), to reduce sedation in light of the patient's reduced agitation.

Over the duration of his admission, the patient eventually became more cooperative, as he improved day by day with his treatment. As discharge approached, the final Lithium and Valproic acid levels were 0.30 mmol/L and 54 mg/L, respectively, both subtherapeutic values. However, due to laboratory issues, the labs were drawn several hours later than ordered, and thus, potentially not reflecting trough levels.

The patient was hospitalized for a total of 12.5 days. A lack of insight was noted during the patient’s hospital stay. He was aware that he had bipolar disorder, and he was aware that his behaviors were concerning. However, he did not fully understand the need for hospitalization. Upon discharge, the patient was psychiatrically stable and did not display any symptoms of psychosis or mania. He also showed a substantial improvement in his thought process. The patient demonstrated that he understood the importance of adhering to his home medication regimen, which was tapered to include risperidone, divalproex sodium, lithium, temazepam, and diphenhydramine (Table 2).

**Table 2:** Discharge medications PO: per os (by mouth). This abbreviation indicates that the medication is taken orally. BID: two times a day. This abbreviation indicates that the medication is taken twice throughout the day. TID: three times a day. This abbreviation indicates that the medication is taken three times throughout the day. PRN: as needed. This abbreviation indicates that the medication is taken only when required. mg: milligrams. This unit of measurement is used to denote the dosage of medications. PRN: when necessary. This abbreviation indicates that the medication was given when required for extenuating circumstances such as excessive agitation.

Discharge medications	Day 12: 10/23/23
Risperidone	2mg PO BID
Divalproex Sodium	500mg PO TID
Lithium	450mg PO BID
Temazapam	30mg PO PRN
Diphenhydramine	50mg PO PRN

## Discussion

Multiple medications were needed to achieve relief of this patient's symptoms, especially considering the high levels of agitation and aggression he displayed. BD-1 is a chronic psychiatric disorder characterized by at least one episode of mania. It is less prevalent among men, with symptoms often beginning in adolescence and significantly impacting a patient's life [4]. Our patient also presented with drug abuse, including MDMA, Cocaine, and Cannabis, with Cannabis being reported as a substance he used daily. This could have exacerbated his mania and psychosis, as the association between BD and substance use disorder (SUD) is an important factor in the prognosis of the disorder [5].

Patients with bipolar are prone to repeat hospitalizations, which can be a burden, financially and otherwise, on both the patient and their family. Those who adhere to their antipsychotic medications have a lower risk of re-hospitalization, but relapse despite appropriate treatment for mania is not uncommon [5]. The unfortunate reality is that patient compliance with medications is not guaranteed, as in this case, we are unable to truly determine if his regimen was ineffective due to noncompliance, abuse of the medications, or abuse of other substances.

It is important to note that mania can lead to brain damage, emphasizing the urgency of effective crisis management [6]. Multiple hospitalizations are often necessary to control these episodes, psychiatric readmissions are common and can be influenced by various factors beyond the disease itself [7]. These factors may include family education about the illness, social environment, discrimination, medication adherence, and financial support. Our patient had 18 hospitalizations in the span of approximately three to four months, a number that is as alarming as it is surprising. Multiple unknown factors likely contributed to this.

Antipsychotic medications such as Olanzapine or Risperidone, when in combination with Lithium, have proved to have a lower risk of re-hospitalization [8]. Our patient presented with severe agitation, leading to the addition of a second antipsychotic and mood stabilizer during hospitalization: olanzapine and lithium, respectively. Our patient was then discharged on the antipsychotic Risperidone, and two mood stabilizers: lithium and divalproex sodium (Table 2). The combination of Risperidone and Lithium specifically, is indicated in the treatment of BD-1, and the goal of this medication regimen was to better control symptoms and to decrease the likelihood of relapse and further hospitalizations [9]. 

The financial and social burden of bipolar disorder can be substantial, with estimates indicating significant lifetime costs associated with the illness [8]. These findings highlight the importance of not only addressing the immediate symptoms of bipolar disorder but also implementing strategies to support patients and their families in navigating the long-term challenges associated with the condition.

Effective crisis management is crucial in the treatment of bipolar disorder, particularly in cases involving multiple hospitalizations. By addressing symptoms promptly and comprehensively, healthcare providers can mitigate the impact of the illness on individuals and their families [6]. Additionally, ongoing support and education are essential for promoting long-term stability and improving outcomes for individuals with bipolar disorder [7]. By addressing the multifaceted challenges associated with the condition, it is possible to enhance the quality of life for those affected and reduce the need for future hospitalizations.

## Conclusions

This report highlights the case of a young, Hispanic male diagnosed with BD-1. This case was unique because it explored challenging symptomatology, leading to a substantial number of hospitalizations. In addition to hard-to-manage symptoms, our patient had a history of substance abuse which may have exacerbated his acute presentations of mania and psychosis. As such, the intention of this report was to highlight the challenges of managing a severely agitated manic patient and the significance of recurrent hospitalizations in such a case of bipolar disorder. This patient was under a medication regimen at the time of admission, but the condition was still not controlled. We hope that with appropriate medication reconciliation and social support, we can help this patient prevent further extensive crises. The purpose of our report is to contribute information that can help mental health providers navigate similar cases, as well as to help the medical community fill knowledge gaps.

## References

[1] Guzman-Parra J, Streit F, Forstner AJ (2021). Clinical and genetic differences between bipolar disorder type 1 and 2 in multiplex families. Transl Psychiatry.

[2] Mehra N, Bhatia A, Ayis S (2024). Reasons for diagnostic delays in bipolar disorder: systematic review and narrative synthesis [PREPRINT]. Qeios.

[3] Diflorio A, Jones I (2010). Is sex important? Gender differences in bipolar disorder. Int Rev Psychiatry.

[4] Dell'Osso B, Cafaro R, Ketter TA (2021). Has bipolar disorder become a predominantly female gender related condition? Analysis of recently published large sample studies. Int J Bipolar Disord.

[5] Li DJ, Lin CH, Wu HC (2018). Factors predicting re-hospitalization for inpatients with bipolar mania--a naturalistic cohort. Psychiatry Res.

[6] Cotovio G, Talmasov D, Barahona-Corrêa JB (2020). Mapping mania symptoms based on focal brain damage. J Clin Invest.

[7] Phillips MS, Steelesmith DL, Campo JV, Pradhan T, Fontanella CA (2020). Factors associated with multiple psychiatric readmissions for youth with mood disorders. J Am Acad Child Adolesc Psychiatry.

[8] Begley CE, Annegers JF, Swann AC, Lewis C, Coan S, Schnapp WB, Bryant-Comstock L (2001). The lifetime cost of bipolar disorder in the US: an estimate for new cases in 1998. Pharmacoeconomics.

[9] McNeil SE, Gibbons JR, Cogburn M (2023). Risperidone. https://www.ncbi.nlm.nih.gov/books/NBK459313/.

